# Characteristics of *Helicobacter pylori* antibiotic resistance: data from four different populations

**DOI:** 10.1186/s13756-019-0632-1

**Published:** 2019-11-27

**Authors:** Dong-sheng Liu, You-hua Wang, Zhen-hua Zhu, Shuang-hong Zhang, Xuan Zhu, Jian-hua Wan, Nong-hua Lu, Yong Xie

**Affiliations:** 1 Department of Gastroenterology, The First Affiliated Hospital of Nanchang University, Nanchang, Jiangxi Province China; 2 Department of Gastroenterology, Children’s Hospital of Jiangxi, Nanchang, Jiangxi Province China

**Keywords:** *Helicobacter pylori*, Clarithromycin, General population, Resistance

## Abstract

**Aims:**

To describe the characteristics of *Helicobacter pylori* (*H. pylori*) antibiotic resistance in clinical isolates from four populations.

**Methods:**

In total, 1463 *H. pylori* strains were examined for antibiotic resistance. Among these strains, 804 were isolated from treatment-naïve adults, 133 from previously treated adults, 100 from treatment-naïve children and 426 from a population who participated in a health survey (age ≥ 40 years). The minimum inhibitory concentration was determined by the E-test method.

**Results:**

In the treatment-naïve adult group, the resistance rates for metronidazole, clarithromycin, levofloxacin, amoxicillin, rifampicin and tetracycline were 78.4, 19.0, 23.3, 1.2, 1.7 and 2.3%, respectively. Compared with this group, the previously treated adult group had significantly higher resistance rates for metronidazole (99.2%), clarithromycin (58.3%) and levofloxacin (52.3%). In addition, the treatment-naïve children had a lower metronidazole resistance rate (46.0%) than the treatment-naïve adults. The resistance rate for clarithromycin was low in treatment-naïve patients with ages ranging from 10 to 24 years. For the strains isolated from the general population group, the resistance rates for metronidazole, clarithromycin, levofloxacin, amoxicillin, rifampicin and tetracycline were 78.6, 10.1, 25.1, 0.5, 2.1 and 0.9%, respectively. Compared with the treatment-naïve adult group, the general population group showed significant differences in clarithromycin resistance.

**Conclusion:**

The resistance rates for metronidazole, clarithromycin and levofloxacin were high, especially in previously treated adults. Compared to those in treatment-naïve younger patients, the resistance rates for clarithromycin were significantly lower in treatment-naïve patients with ages ranging from 10 to 24 years and in the general population.

## Introduction

*Helicobacter pylori* (*H. pylori*) is the major pathologic agent in the development of gastritis, peptic ulcers, atrophic gastritis and gastric adenocarcinoma [1, 2]. Although the prevalence of *H. pylori* infection is decreasing in some western countries, the situation remains severe in some Asian countries, where more than half of the population is infected by *H. pylori* [3–5]. The large infected population makes resolving this issue an urgent problem. Antibiotic resistance is a key factor that induces anti-*H. pylori* treatment failure [6, 7]. Strains resistant to clarithromycin show a significantly lower eradication rate when treated with clarithromycin-based therapies [8], and the eradication rates in strains with gyrA mutations were half of those in strains without gyrA mutations when levofloxacin-based treatments were applied [6, 7]. In addition, unsuccessful prior eradication attempts were proved to be a major risk factor for resistance development [9]. Tailored treatment is a promising therapeutic approach to achieve a good eradication rate [10, 11], but most areas still perform empirical treatment, such as bismuth-containing quadruple or concomitant therapies, as individual antibiotic resistance characteristics are more difficult to determine; among these empirical therapies, metronidazole, clarithromycin, levofloxacin and amoxicillin are widely used.

While empirical treatment is widely performed, it is crucial to understand how antibiotic resistance is different among different populations. Among Israeli children and adolescents, compared with patients naïve to anti-*H. pylori* treatment, patients who were previously treated for *H. pylori* had a threefold higher resistance rate for clarithromycin and a twofold higher resistance rate for metronidazole [12]. The resistance rates for metronidazole and ciprofloxacin were significantly higher in adults than in children [13]. In addition, the antibiotic resistance rate is also associated with age, gender, the type of gastric disease, and the location of the patient [14, 15]. Most studies have focused on patients in hospitals, and the prevalence of antibiotic resistance in the general population remains unclear; in addition, most studies reported resistance rate changes in only adults or children, and fewer studies reported resistance changes in both populations. To address these problems, we performed this study.

The present retrospective study was designed to evaluate the prevalence of antibiotic resistance in *H. pylori* isolates from adults with or without prior anti-*H. pylori* treatment, treatment-naïve children, and a population who participated in a health survey.

## Materials and methods

### Patients and strains

A retrospective study was conducted on *H. pylori*-infected patients with positive *H. pylori* cultures between 2010 and 2017 at the First Affiliated Hospital of Nanchang University, Jiangxi Province. From these patients, a total of 1463 *H. pylori* isolates (one isolate per patient) were obtained from gastric biopsy specimens.

Based on the characteristics of the patients, four groups were created. The first group was composed of adult patients (age ≥ 18 years) who were naïve to anti-*H. pylori* treatment; The second group was composed of adult patients (age ≥ 18) who had previously received treatment for *H. pylori* infection and confirmed eradication failure within 4–8 weeks after anti-*H. pylori* treatment was completed. Patients in these two groups were enrolled at the First Affiliated Hospital of Nanchang University, Jiangxi Province. The third group was composed of child patients (age < 18) who were naïve to anti-*H. pylori* treatment; the patients in this group were enrolled at the First Affiliated Hospital of Nanchang University and the Children’s Hospital of Jiangxi Province. The fourth group was composed of individuals who completed a health survey performed in the general population (age ≥ 40) in Yudu County, Jiangxi Province, China, regardless of the presence of digestive symptoms. When the serology test suggested *H. pylori* infection, gastroscopy was performed to obtain a biopsy for the culture of *H. pylori*. The protocol was approved by the Ethics Committee of The First Affiliated Hospital of Nanchang University (2018085).

### *H. pylori* culture and antibiotic susceptibility test

Briefly, gastric mucosal biopsy specimens from the antrum and corpus were stored in brain heart infusion broth (Oxoid, Basingstoke, UK) with 20% glycerin at − 80 °C before use. After homogenization, the biopsies were cultured on Campylobacter agar (Oxoid, Basingstoke, UK) plates supplemented with 5% defibrinated sheep blood (Bio-Kont, Zhejiang, China), 2.5 mg/L vancomycin, 3 mg/L trimethoprim, 2 mg/L polymyxin B and 2 mg/L amphotericin B (Duly Biotech, Nanjing, China). The plates were incubated in a microaerobic atmosphere (10% CO_2_, 5% O_2_ and 85% N_2_) at 37 °C for up to 5 days.

Susceptibility to 6 antibiotics (metronidazole, clarithromycin, levofloxacin, amoxicillin, rifampicin and tetracycline) was tested via the E-test method. The resistance break points for metronidazole, clarithromycin, levofloxacin, amoxicillin, rifampicin and tetracycline were set at > 8, > 0.5, > 1, > 1, > 1 and 12 mg/L, respectively (http://www.eucast.org/fileadmin/src/media/PDFs/EUCAST_files/Breakpoint_tables/Breakpoint_table_v_3.1.pdf). *H. pylori* culture and antibiotic susceptibility testing were performed by the Institute of Gastroenterology and Hepatology of the First Affiliated Hospital of Nanchang University.

### Statistical analysis

All statistical analyses were carried out using SPSS statistical software (version 20; SPSS, Chicago, USA). Differences in resistance rates among different groups were assessed with the chi-squared test or Fisher’s exact test. A two-tailed *P* value less than 0.05 was considered statistically significant.

## Results

In total, 1463 strains were included in the analysis. Among these strains, 804 were isolated from anti-*H. pylori* treatment-naïve adult patients (mean age 40.1 years), 133 were isolated from previously treated adult patients (mean age 47.4 years), 100 strains were isolated from anti-*H. pylori* treatment-naïve children (mean age 9.6 years), and 426 strains were isolated from individuals in the general population who participated in a health survey (mean age 53.1 years).

### (a) Comparison of resistance rates for strains from treatment-naïve and previously treated patients

**Fig. 1:**
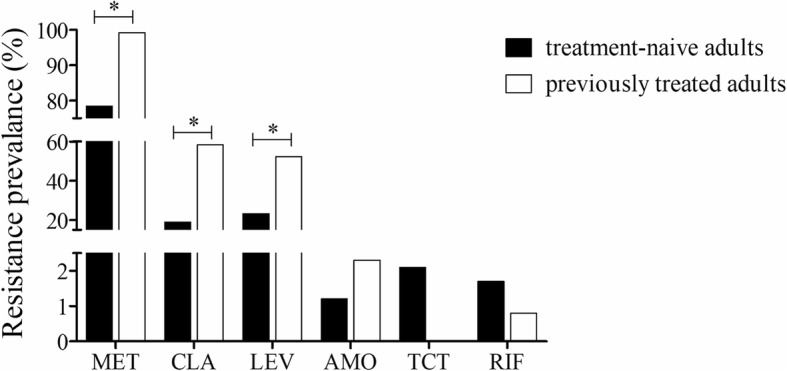
Resistance rates for strains isolated from patients who were anti-*H. pylori* treatment-naïve or had previously received treatment. (MET, metronidazole; CLA, clarithromycin; LEV, levofloxacin; AMO, amoxicillin; TCT, tetracycline; RIF, rifampicin. *: *p* < 0.05)

Compared with strains isolated from treatment-naïve adult patients, strains isolated from previously treated adult patients had significantly higher resistance rates for metronidazole (99.2% vs 78.4%, *P* < 0.001), clarithromycin (58.3% vs 19.0%, *P* < 0.001), and levofloxacin (52.3% vs 23.3%, *P* < 0.001). There were no significant differences in resistance rates for amoxicillin (2.3% vs 1.2%, *P* = 0.401), rifampicin (0.8% vs 1.7%, *P* = 0.708) or tetracycline (0.0% vs 2.3%, *P* = 0.092) between these two groups (Fig. 1).

### (b) Comparison of resistance rates for strains from treatment-naïve adult and child patients

**Fig. 2:**
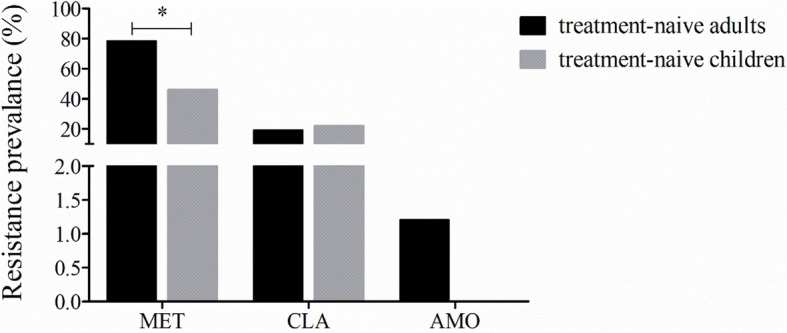
Resistance rates for strains from treatment-naïve adult and pediatric patients. (MET, metronidazole; CLA, clarithromycin; AMO, amoxicillin. *: *p* < 0.05)

**Fig. 3:**
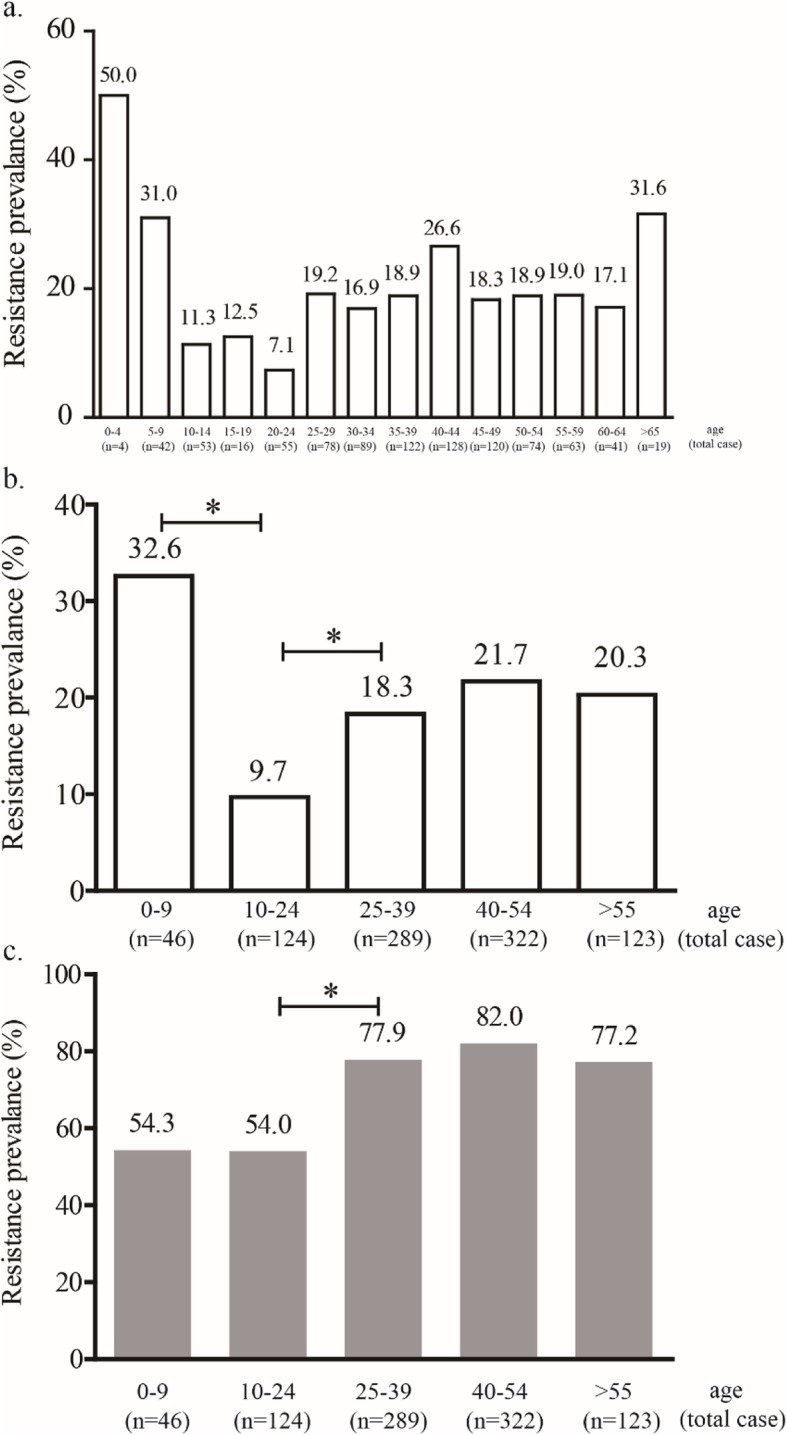
The association between age and resistance rates. **a** The prevalence of clarithromycin resistance in patients divided into 5-year age classes; **b** The prevalence of clarithromycin resistance in patients divided into 15-year age classes; and **c** The prevalence of metronidazole resistance in patients divided into 15-year age classes

For the strains isolated from children, we detected susceptibility to amoxicillin, metronidazole and clarithromycin. Compared with strains isolated from treatment-naïve adult patients, strains isolated from treatment-naïve children had a lower resistance rate for metronidazole (46.0% vs 78.4%, *P* < 0.001), but there were no significant differences in the resistance rates for amoxicillin (0.0% vs 1.2%, *P* = 0.613) or clarithromycin (22.0% vs 19.0%, *P* = 0.502) (Fig. 2). However, further analysis of the relationship between age and the clarithromycin resistance rate showed significant differences among different age groups, with a higher clarithromycin resistance rate in patients aged 5–9 years than in patients aged 10–14, 15–19 or 20–24 years (Fig. 3a). When the groups were combined, the results showed a lower clarithromycin resistance rate in patients aged 10–24 years than in patients aged 0–9 years (9.7% vs 32.6%, *P* < 0.001) and patients aged 25–39 years (9.7% vs 18.3%, *P* = 0.027) (Fig. 3b). We also performed a subgroup analysis for metronidazole resistance, and the results showed that compared to that in patients aged 10–24 years, the resistance rate was significantly higher in patients aged 25–39 years (54.0% vs 77.9%, *P* < 0.001), but no significant difference was observed compared to that in patients aged 0–9 years (54.0% vs 54.3%, *P* = 0.971) (Fig. 3c).

### (c) Comparison of resistance rates for strains from treatment-naïve adult patients and individuals in the general population who participated in a health survey

**Fig. 4:**
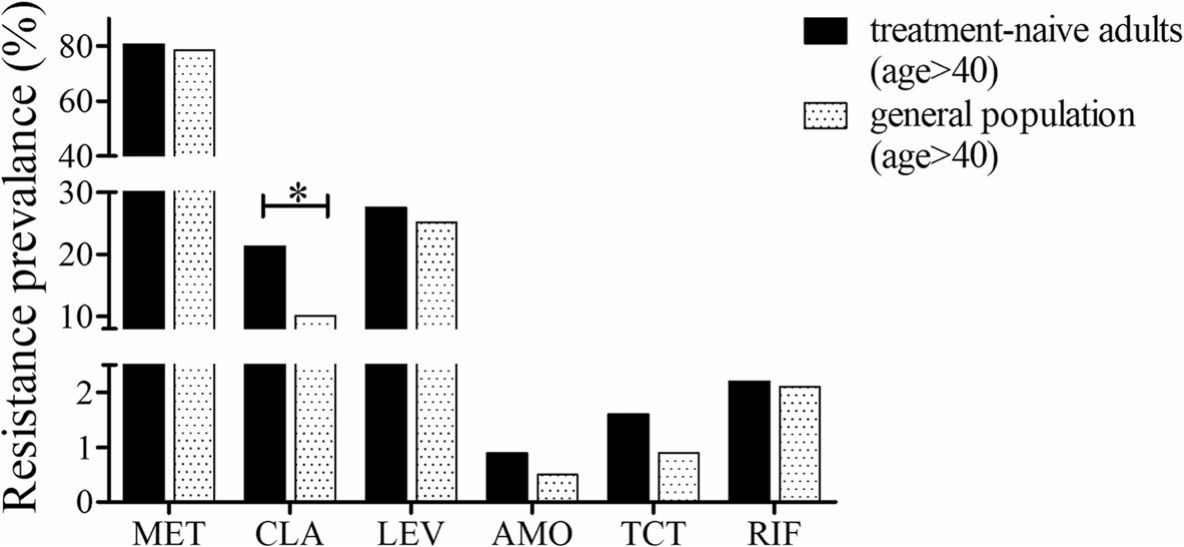
Resistance rates for strains from treatment-naïve adult patients and individuals in the general population who participated in a health survey. (MET, metronidazole; CLA, clarithromycin; LEV, levofloxacin; AMO, amoxicillin; TCT, tetracycline; RIF, rifampicin. *: *p* < 0.05)

The individuals who participated in the health survey were required to be over 40 years old. As age is a factor related to antibiotic resistance, this population was compared with only those treatment-naïve adults who were also over 40 years old. Compared with strains isolated from treatment-naïve adult patients, strains isolated from the general population who participated in the health survey had lower resistance rates for clarithromycin (10.1% vs 21.3%, *P* < 0.001), while there were no significant differences between the two groups when comparing the resistance rates for metronidazole (78.6% vs 80.7%, *P* = 0.501), levofloxacin (25.1% vs 27.6%, *P* = 0.443), amoxicillin (0.5% vs 0.9%, *P* = 0.687), tetracycline (0.9% vs 1.6%, *P* = 0.547) and rifampicin (2.1% vs 2.2%, *P* = 1.000) (Fig. 4).

## Discussion

The present retrospective study was performed to evaluate the prevalence of resistance to different antibiotics in *H. pylori* isolates. The results revealed that there is high primary resistance to metronidazole, clarithromycin and levofloxacin. In addition, the resistance rates were higher in strains isolated from patients who had received prior anti-*H. pylori* treatment, especially for metronidazole, as the resistance rates against metronidazole were almost hundred percent. As we could not obtain data on previous antibiotic susceptibility, it was difficult to clarify whether these high resistance rates were induced or filtered by the anti-*H. pylori* treatment. These data also suggest that the use of these antibiotics for these patients without individual antibiotic susceptibility data carries a high risk. These results were consistent with those of Wueppenhorst et al., who found that compared with strains from treatment-naïve patients, strains from previously treated patients showed significantly higher resistance to metronidazole, clarithromycin, and levofloxacin [9]. Similar results were also found in children and adolescent patients [12].

We also performed a comparison between treatment-naïve adults and children, and we found a significant difference in the metronidazole resistance rates between the two groups. Similar results were also observed in Bulgarian patients [16]. This may be caused by the use of metronidazole in adults to treat oral or gynecological disease [17, 18]. While no significant difference in clarithromycin resistance rates was found between these two groups, the resistance rate for clarithromycin appeared to be somewhat higher in children than in adults; this phenomenon was also observed in other studies [19–21]. When we performed further analysis to evaluate the association between age and the clarithromycin resistance rate, patients under the age of 10 years had a significantly higher resistance rate than patients with ages ranging from 10 to 24 years; after the age of 24 years, the resistance rate rose again. It is possible that young children have a higher resistance rate to clarithromycin because macrolides are widely used for respiratory infections such as *mycoplasma pneumonia* in childhood. Interestingly, the resistance rate decreased in the following age class. A downward trend in clarithromycin resistance was also observed in research by L Li [22]. It appears that resistance to clarithromycin is reversible or maybe resistant strains are replaced by susceptible strains. It has been reported that without the pressure of antibiotics, susceptible bacteria are able to outcompete resistant strains over time, as bacteria may incur a fitness cost to acquire resistance [23]. This may account for the change in clarithromycin resistance rates. In addition, we detected resistance to only three antibiotics in *H. pylori* strains isolated from child patients because the use of antibiotics such as levofloxacin and tetracycline is limited in child patients. Quinolone antibiotics such as levofloxacin may have some bad effects on the development of bone and cartilage in children, and the use of tetracycline can induce tetracycline pigmentation of teeth.

In addition, we also collected strains from the general population; to the best of our knowledge, this was the first study to focus on this population group. This group was evaluated to determine the prevalence of antibiotic resistance regardless of the presence of digestive symptoms or previous treatment. The results showed that there were no significant differences when comparing the resistance rates for metronidazole, levofloxacin, amoxicillin and other antibiotics between the general population and the treatment-naïve adult population. It is noteworthy that again, the result differs for clarithromycin, whose resistance rates were significantly lower in this group than in the treatment-naïve adult group. Approximately 10% of *H. pylori*-infected patients have digestive symptoms, while most *H. pylori*-positive patients do not report any discomfort. Most of the general population belongs to this latter category, while patients who seek treatment in the hospital have digestive symptoms. It is possible that these patients had a higher level of macrolide consumption than the general population before going to the hospital. It should be noted that only clarithromycin resistance showed a possible reversal phenomenon, suggesting some unique characteristic of this antibiotic.

The current study had some limitations. First, we detected only three antibiotic resistance rates in treatment-naïve children, making the comparison incomplete. Second, we were unable to obtain the treatment protocols and original resistance rates for the previously treated adult patients. Third, the individuals in the general population who participated in the health survey were all older than 40 years, so some bias may exist.

## Conclusions

The resistance rates for metronidazole, clarithromycin and levofloxacin were high, especially in previously treated adults. Treatment-naïve children had a lower metronidazole resistance rate than treatment-naïve adults. The resistance rate for clarithromycin was low in treatment-naïve patients whose ages ranged from 10 to 24 years. The resistance rates for clarithromycin were significantly lower in the general population than in treatment-naïve adult patients.

## Data Availability

Data sharing not applicable to this article.

## Abbreviations

E-test: Epsilometer test
*H. pylori*: *Helicobacter pylori*
